# Novel Isocyanate-Modified Carrageenan Polymer Materials: Preparation, Characterization and Application Adsorbent Materials of Pharmaceuticals

**DOI:** 10.3390/polym9110595

**Published:** 2017-11-10

**Authors:** Myrsini Papageorgiou, Stavroula G. Nanaki, George Z. Kyzas, Christina Koulouktsi, Dimitrios N. Bikiaris, Dimitra A. Lambropoulou

**Affiliations:** 1 Laboratory of Environmental Pollution Control, Department of Chemistry, Aristotle University of Thessaloniki, GR-541 24 Thessaloniki, Greece; myrsinipapag@gmail.com; 2 Laboratory of Polymer Chemistry and Technology, Department of Chemistry, Aristotle University of Thessaloniki, GR-541 24 Thessaloniki, Greece; sgnanaki@chem.auth.gr (S.G.N.); ckoulouktsi@pharmathen.com (C.K.); dbic@chem.auth.gr (D.N.B.); 3 Hephaestus Advanced Laboratory, Eastern Macedonia and Thrace Institute of Technology, GR-654 04 Kavala, Greece; georgekyzas@gmail.com

**Keywords:** carbamazepine, carrageenan polymers, diclofenac, dispersive solid phase extraction, response surface methodology

## Abstract

The present study focused on the synthesis and application of novel isocyanate-modified carrageenan polymers as sorbent materials for pre-concentration and removal of diclofenac (DCF) and carbamazepine (CBZ) in different aqueous matrices (surface waters and wastewaters). The polymer materials were characterized using Fourier transform infrared spectroscopy (FTIR), X-ray diffraction (XRD), Thermal Gravimetric Analysis (TGA) and Scanning Electron Microscopy (SEM). The effects on the adsorption behavior were studied, and the equilibrium data were fitted by the Langmuir and Freundlich models. The maximum adsorption capacity (*Q_max_*) was determined by Langmuir–Freundlich model and was ranged for iota-carrageenan (iCAR) from 7.44 to 8.51 mg/g for CBZ and 23.41 to 35.78 mg/g for DCF and for kappa-carrageenan (kCAR) from 7.07 to 13.78 mg/g for CBZ and 22.66 to 49.29 mg/g for DCF. In the next step, dispersive solid phase extraction (D-SPE) methodology followed by liquid desorption and liquid chromatography mass spectrometry (LC/MS) has been developed and validated. The factors, which affect the performance of D-SPE, were investigated. Then, the optimization of extraction time, sorbent mass and eluent’s volume was carried out using a central composite design (CCD) and response surface methodology (RSM). Under the optimized conditions, good linear relationships have been achieved with the correlation coefficient (*R*^2^) varying from 0.9901 to 0.995. The limits of detections (LODs) and limits of quantifications (LOQs) ranged 0.042–0.090 μg/L and 0.137–0.298 μg/L, respectively. The results of the recoveries were 70–108% for both analytes, while the precisions were 2.8–17.5% were obtained, which indicated that the method was suitable for the analysis of both compounds in aqueous matrices.

## 1. Introduction

Detection of pharmaceutically active compounds in the aquatic environment has raised concerns over their potential adverse effects on the environment [1,2,3]. Among them, carbamazepine (CBZ), a well-known antiepileptic compound, and diclofenac (DCF), a common non-steroidal anti-inflammatory drug, owing to ever-increasing consumption, inappropriate disposal and their incomplete removal in wastewater treatment plants (WWTPs) have been found ubiquitously in wastewater effluents [4,5,6] and different waters including surface water, ground water, and drinking water worldwide [7]. The release rate of CBZ into water bodies is estimated to be around 30 tons per year and, therefore, CBZ has been proposed as an anthropogenic marker for water contamination in the environment. According to several reports, environmentally-relevant concentrations of CBZ could negatively influence aquatic life (i.e., bacteria, algae, invertebrates, and fish) [8,9]. On the other hand, the anti-inflammatory DCF was identified for priority investigation because of risk perception and has recently been included on the “watch list” of priority substances under the Water Framework Directive [10]. The global consumption of DCF is estimated to be 940 tons per year, with a defined daily dose of 100 mg [9,11]. 

These facts have drawn extensive interest into the investigation of both compounds. Hence, it is important to develop reliable techniques for the detection and quantification of trace concentrations of these compounds in water samples in order to assess environmental exposure to CBZ and DCF. In general, the quantification of pharmaceutical compounds is usually carried out with chromatographic techniques after their extraction from aqueous matrices with a suitable sample preparation method. Among the various sample preparation methods, sorptive microextraction techniques (SμE) (e.g., Solid Phase Microextraction (SPME); Dispersive solid phase extraction, etc.) [12,13,14,15,16], for pre-concentration and/or cleanup of drugs offer new possibilities in sample treatment and superior advantages compared to conventional extraction methods. Accordingly, novel sorptive microextraction methods are being developed and examined for extraction of pharmaceuticals and other emerging contaminants from aqueous media with a focus on new materials with remarkable properties. The application of new sorbents has achieved a sharp increase in recent years since they can play an important role in sample and pre-concentration processes [17,18,19,20,21,22].

Given this background, in the present study, two isocyanate-modified carrageenan polymers based on kappa-carrageenan (kCAR) and iota-carrageenan (iCAR) have been synthesized and evaluated as sorbents for extraction of CBZ and DCF. Due to non-toxicity, biodegradability, and biocompatibility of carrageenan biopolymers there are considerable research efforts to provide new carrageenan-based materials [23]. In our previous article, iCAR and kCAR carrageenan microparticles were synthesized by using glutaraldehyde as cross-linking agent and successfully applied as biosorbents for the removal of the beta blocker metoprolol from aqueous solutions [24]. To move a step forward, in this study, two new carrageenan polymers modified by toluene-2,4-diisocyante were prepared and tested as sorbent materials for D-SPE determination of two of the most frequently detected pharmaceutical compounds. The strong novelty of this work is that this is the first report in which the carrageenan polymers have been used as extraction sorbents for determination of pharmaceuticals in aqueous media. The combination of the polymers synthesis (characterizations, etc.) along with their application as extraction sorbents, especially in the case of a very sensitive class of environmental pollutants as pharmaceuticals, provides an extra novelty to this work.

## 2. Materials and Methods 

### 2.1. Materials

iCAR (Gelcarin GP-379NF) and kCAR (Gelcarin GP-812NF) were kindly supplied by FMC BioPolymer (FMC BioPolymer, Vlijmen, The Netherlands). Toluene-2,4-diisocyante (assay 95%) was purchased from Sigma-Aldrich (Sigma-Aldrich, St. Louis, MO, USA). Carbamazepine (CBZ; purity ≥ 99.0%) was purchased from Acros Organics (Acros Organics, Morris Plains, NJ, USA). Diclofenac sodium (DCF; purity ≥ 99.0%) was purchased from Sigma-Aldrich (Table 1). All other and solvents used were of analytical grade, and those used for HPLC analysis were of HPLC grade. 

### 2.2. Synthesis of Isocyanate-Functionalized Carrageenans (CAR-TDI)

iCAR or kCAR (15 g), respectively, was inserted in round-bottomed flask containing 50 mL of dimethylsulfoxide and stirred at room temperature until suspension was formed. Then, 100 mL of tolylene diisocyanate (TDI) was inserted into the flask, and vigorous stirring was performed at 50 °C for 4 h. The formed gel was cut into pieces and soaked in 100 mL of dichloromethane under magnetic stirring for 2 h in order to remove dimethylsulfoxide and unreacted TDI. Further purification was also done by inserting the material in distilled water at 80 °C for 3 h (for the removal of unreacted carrageenan) and finally washing with ethanol. The synthesized material was left to dry under vacuum. The prepared isocyanate-functionalized iota- (iCAR-TDI) and kappa- (kCAR-TDI) carrageenans were further chopped using cutter mill and particle size of about 100 μm was taken and stored in a desiccator till further use. 

### 2.3. Characterization Techniques

Fourier transform infrared spectroscopy (FTIR) was used to characterize the functionalized carrageenans and possible bonds formed during drug adsorption experiments. The spectra were collected using a Perkin-Elmer FTIR spectrometer (model FTIR-2000, Perkin Elmer, Dresden, Germany). In brief, 5 mg of each sample was mixed with 180 mg of KBr in an agate mortal. The mixture was pressed under 5 tons for 2 min and pellet was formed. The pellet was then placed into an attachment in the optical compartment and FTIR spectra were obtained using. Infrared (IR) absorbance spectra were obtained between 450 and 4000 cm^−1^ at a resolution of 4 cm^−1^ using 20 co-added scans. All spectra submitted to baseline correction and normalization to 1. 

X-ray diffraction (XRD) patterns were taken using a Rigaku Mini Flex diffractometer with Bragg-Brentano geometry (θ, 2θ) and a Ni-filtered CuKα radiation. Analysis was performed on net carrageenans, functionalized carrageenans with TDI and drug adsorbed samples. The samples were scanned over the internal range of 5–60°, step 0.02°, speed 2.0°/min.

Thermal Gravimetric Analysis (TGA) analysis was carried out with a SETARAM SETSYS TG-DTA 16/18. Samples (6.0 ± 0.2 mg) were placed in alumina crucibles. An empty alumina crucible was used as reference. Net and functionalized carrageenans were heated from ambient temperature to 800 °C in a 50 mL/min flow of N_2_ at heating rates of 20 °C/min. Continuous recordings of sample temperature, sample weight, its first derivative and heat flow were performed.

### 2.4. Chromatographic Analysis

The Liquid Chromatographic (LC) system consisted of a SIL 20A autosampler with the volume injection set to 20 μL and LC-20AB pump both from Shimadzu (Kyoto, Japan). Chromatographic separation was achieved using a C_18_ (Athena) analytical column 250 × 4.6 mm with 5 μm particle size. Detection was performed using a SPD 20A DAD detector coupled in series with the LC-MS 2010EV mass selective detector, equipped with an atmospheric pressure electrospray ionization (ESI) source. The samples were analyzed using the ESI interface in positive ionization (PI) mode for CBZ and in negative mode (NI) for DCF. The mobile phase consisted of water with 0.1% formic acid (A) and methanol (B) in isocratic elution program (10% A:90% B). Column temperature was set at 40 °C and the flow rate was 0.4 mL/min. The drying gas was operated at flow 10 L/min at 200 °C. The nebulizing pressure was 100 psi, capillary voltage was 4500 V for positive ionization and −3500 V for negative ionization and the fragmentation voltage was set at 5 V. For each compound, the precursor molecular ion in the selected-ion monitoring (SIM) mode was acquired ([M + H] 237 *m*/*z* for CBZ and [M − H] 294 *m*/*z* for DCF). 

### 2.5. Adsorption Experimental Procedure

The adsorption evaluation was done running batch experiments (all experiments were run in triplicate). However, some slight differences in experimental conditions for the adsorption of CBZ and DCF changed the whole experimental adsorption design. The latter was done due to the differences of chemical structures of model pollutants and as a result differences in p*K*a, solubility, decomposition, etc.

In the case of CBZ adsorption experiments, 1 g of adsorbent material was used per 1 L of aqueous solution (*m* = 0.030 g of adsorbent’s mass were added to *V* = 30 mL of deionized water in a conical flask). In particular, samples were taken at predetermined time intervals and filtered using 45 μm pore size filtration membrane (Whatman, purchased by Sigma-Aldrich). However, some slight differences in experimental conditions for the adsorption of CBZ and DCF occurred. For the pH-effect experiments (*C*_0_ = 30 mg/L), the solution pH was initially adjusted with aqueous solutions of acid or base (0.01 mol/L of HCl and/or 0.01 mol/L NaOH) to reach the appropriate pH values (2–10 for CBZ and 6–12 for DCF). The agitation rate was fixed at *N* = 150 rpm for all adsorption-desorption tests using shaking incubator (model Grant Instruments OLS Aqua Pro, Cambridge, UK) under a controlled temperature. Isotherms were taken running the adsorption experiments with various initial drug concentrations (pH = 2, 10; *C*_0_ = 0–100 mg/L) at *T* = 20, 30, 40 °C for 24 h (contact time) (Table 2).

In the case of DCF adsorption experiments, 0.5 g of adsorbent material was used per 1 L of aqueous solution (*m* = 0.015 g of adsorbent’s mass were added to *V* = 30 mL of deionized water in a conical flask). All other conditions were kept the same: *C*_0_ = 30 mg/L for pH-effect experiments; *N* = 150 rpm. Isotherms were carried out (pH = 6) at *T* = 20, 30, 40 °C for 24 h (contact time) for varying initial DCF concentrations (*C*_0_ = 0–70 mg/L). The value of pH (pH = 6) for carrying out isothermal experiments was selected as it was found to be the optimum value according to pH-effect tests (Table 2). 

### 2.6. Dispersive Solid Phase Extraction Procedure (D-SPE)

The D-SPE procedure was commenced by adding 15 mg of the sorbent to 15 mL of pH adjusted sample (CBZ: pH = 10; DCF: pH = 6). A suspension was then mechanically shaken at 1000 rpm for 15 min to allow sorption of the analytes onto the sorbent. After that the suspension was centrifuged at 5000 rpm for 10 min, and the supernatant was discarded. Then, a desorption procedure was carried out as follows: 1 mL methanol was added into the vial and the analytes were desorbed via ultrasonic treatment for 10 min. Then, the supernatant was collected and evaporated to dryness under a gentle stream of nitrogen gas. Finally, the extract was re-dissolved in 50 μL of the mobile phase and injected into the LC-MS system. 

### 2.7. Validation Study, Quality Assurance/Quality Control 

Validation of the method was performed according to Document No. SANTE/11945/2015 [25]. The parameters: linearity, limits of detection (LOD), limits of quantification (LOQ), recovery, precision and uncertainty were evaluated. Selectivity of the method was estimated considering the absence of interfering peaks at the retention time of each compound. The linearity of the method was determined under optimized experimental conditions with pure solvents and matrix-matched standards (*n* = 5). Calibration curves were fitted by least-square regression and linearity was assumed when correlation coefficient (*r*^2^) was higher than 0.990 with residuals lower than 20%.Responses in solvent and in matrix were compared to evaluate the matrix effect. The LOQs were defined as the minimum concentration of the analyte that can be quantified with acceptable recovery (in range 70–120%) and precision (RSD ≤ 20%). LOD were calculated using signal-to-noise ratio (S/N) criteria, in all cases; LOD = 3 S/N.

Accuracy and precision of the method were tested by means of recovery experiments, performed with six replicates of blank samples spiked with the target pharmaceuticals at 1, 10 and 100 μg/L. The recovery was determined by means of the measured concentration versus the spiked concentration. The mean recoveries and corresponding relative standard deviation (RSD) were calculated for accuracy and precision evaluation at three different concentration levels, corresponding to the LOQ level, to a medium and to a high concentration level of the calibration curves. Intra-day assays were performed by spiking blanks using six replicates for each concentration level in one day. Six replicates were performed for each level on six consecutive days under within-laboratory reproducibility conditions. Acceptable mean recoveries are those within the range 70–120%, with an associated repeatability RSD ≤ 20%, for all analytes within the scope of a method [10]. Blank samples also undergo the same procedure as with the real sample analysis. 

Finally, a quality assurance and quality control were also performed. Procedural blanks were injected to monitor for background contamination. These blanks were processed in the same way as the samples and injected into the LC-MS system. Target compounds were not determined above LOQs. On the other hand, in order to validate both the calibration and the method stability, a quality control standard at an intermediate concentration was extracted and analyzed in each set of analysis.

Uncertainties were estimated on the basis of in-house validation data according to EURACHEM/CITAC [26,27,28], Eurolab [29,30] and Expression of Uncertainty in Measurement (GUM) guidelines [31], at three spiking levels, as it was described in our previous works [13,15]. Uncertainties were assessed for different water matrices spiked at three concentration levels. The expanded uncertainty (U) was calculated by using the coverage factor *k* = 2, at the confidence level of 95%.

### 2.8. Central Composite Design

Response surface methodology (RSM), as an effective statistical model, has been widely used for optimization of sample preparation methods [15]. In this study, a three-level factor, Central Composite Design (CCD) (*a* = 1.681, rotatable) was applied to optimize extraction conditions for both target analytes. The design comprises 17 trials including 3 at central point, 6 at axial and 8 at factorial point from three independent variables (extraction time (A), sorbent mass (B), and eluent volume (C)) at three levels of the system. The dependent and independent variables, with their low, medium and high levels were selected based on the results from preliminary experiments and according to the literature reports and instrumental aspects. All experiments were performed in a random manner to avoid any systematic bias in the outcomes. Two replicates were performed and the averages of results were taken as a response. The main factors, their symbols, levels and design matrix as well as the response of each run are shown in Table 3. 

A program Design Expert (Trial version 7.0.0 Stat-Ease, Inc., Minneapolis, MN, USA) was applied for regression analysis of the data obtained and to estimate the response function. A quadratic model was built to describe a relationship between the DCF and CBZ extraction percent and operating parameters as defined by Equation (1):

(1) $$Y=\beta_{0}+\sum_{j=1}^{k}\beta_{j}X_{j}+\sum_{j=1}^{k}\beta_{jj}X_{j}^{2}+\sum_{i}\sum_{<j=2}^{k}\beta_{ij}X_{i}X_{j}+e_{i}$$

where *Y* is the response variable, *X_i_* and *X_j_* are the independent variables, and *k* is the number of tested variables (*k* = 3). Regression coefficient is defined as *β*_0_ for intercept, *β_j_* for linear, *β_jj_* for quadratic, *β_ij_* for cross product term and *e_i_* represents the residual term.

The method of signals and the analysis of variance (ANOVA) (95% confidence level) were used to estimate the significant main effects and interactions of factors (Table 4 and Table 5). 

The significance of each coefficient and the interaction between each independent variable were evaluated according to the *p*-value at the 5% significance level. The adequacy of the model was verified using the determination coefficient *R*^2^, the adjusted determination coefficient *R*^2^ and the lack of fit test. 

The fitted polynomial equation is expressed as surface and contour plots in order to visualize the relationship between the response and experimental levels of each factor and to evaluate the optimum conditions. Desirability function was used for simultaneous optimization of all affecting parameters in order to achieve the highest extraction efficiency (%). The adequacy of the model equation for predicting the optimum response values was validated with experimental results constructed to evaluate the optimum conditions for the response variables.

## 3. Results

### 3.1. Characterization of Materials

**Figure 1:**
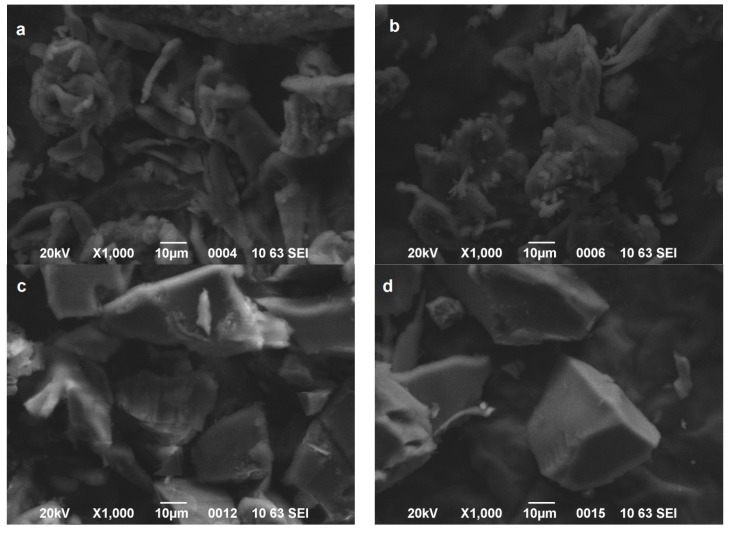
Scanning Electron Microscopy (SEM) images of: (**a**) kCAR; (**b**) iCAR; (**c**) kCAR-TDI; and (**d**) iCAR-TDI.

The morphology of the prepared materials was examined by SEM images. It was found that kCAR and iCAR have smooth surfaces without any specific shape (Figure 1a,b). The modification with TDI caused more rigid and hard surface (Figure 1c,d), while after chopping in cutting mill, the particles retained an irregular shape with sharp edges. This is an indication that the reaction had taken place.

**Figure 2:**
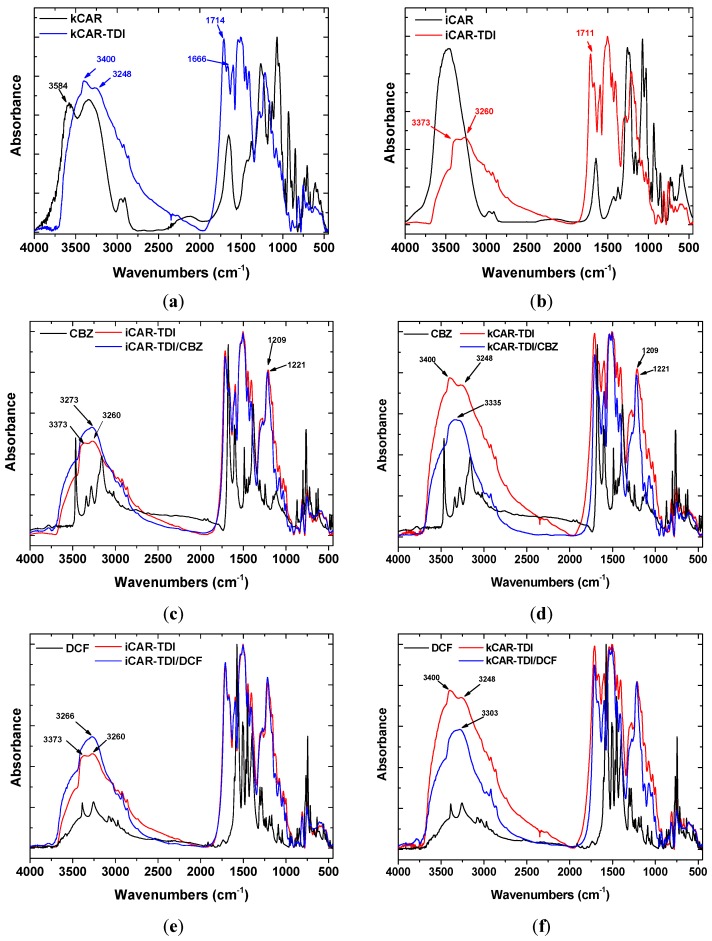
Fourier transform infrared spectroscopy (FTIR) spectra of iCAR and kCAR before and after drugs adsorption. (**a**) black line: kCAR; blue line: kCAR-TDI; (**b**) black line: iCAR; red line: iCAR-TDI; (**c**) black line: CBZ; red line: iCAR-TDI; blue line: iCAR-TDI after CBZ adsorption; (**d**) black line: CBZ; red line: kCAR-TDI; blue line: kCAR-TDI after CBZ adsorption; (**e**) black line: DCF; red line: iCAR-TDI; blue line: iCAR-TDI after DCF adsorption; (**f**) black line: DCF; red line: kCAR-TDI; blue line: kCAR-TDI after DCF adsorption.

Figure 2a,b shows the FTIR spectra of neat and modified carrageenans before adsorption of CBZ, DCF. All the characteristic peaks of net carrageenans are present in the spectra: 3600–3200 cm^−1^ (broad peak owing to O–H interactions), 1645–1755 cm^−1^ (carbonyl group stretching), 1267 cm^−1^ (O=S=O asymmetric stretching), 1156 cm^−1^ (C–O–C asymmetric stretching), 1067–1074 cm^−1^ (S–O symmetric stretching), 1027–1038 cm^−1^ (C–O stretching), 928–935 cm^−1^ (C–O–C stretching in 3,6-anhydrogalactose) and 852 cm^−1^ (C–O–S) stretching in a (1-3)-d-galactose [32]. FTIR spectra of modified carrageenans are also present in Figure 2. It was observed that kCAR-TDI (Figure 2a) formed a new peak at 3248 cm^−1^ owing to >N–H stretching vibrations. In advance, two new peaks were also recorded at 1666 and 1714 cm^−1^ owing to >C=O stretching vibration. In addition, the absence of peak at 2275 cm^−1^ (–N=C=O group) confirmed the absence of unreacted TDI. Similar findings were also presented for iCAR-TDI with >Ν–H group to be recorded at 3260 cm^−1^ (Figure 2b).

Before analyzing the loaded-adsorbents (after adsorption of CBZ or DCF), it is necessary to present the FTIR of each drug separately. CBZ spectrum corresponds to those previously reported for the polymorph form III. Characteristic peaks were observed at 3461 (−NH valence vibration), 1676 (−CO−R vibration), 1594 and 1602 cm^−1^ (in the range of −C=C− and −C=O vibrations; −NH deformation). On the other hand, DCF spectrum exhibited distinctive peaks at 3381 cm^−1^ due to N−H stretching of the secondary amine, 1571 cm^−1^ owing to –C=O stretching of the carboxyl ion and at 748 cm^−1^ owing to C−Cl stretching.

After adsorption, there were shifts of wavenumbers. For CBZ adsorption, in the case of iCAR-TDI (Figure 2c), a shift from 3260 to 3273 cm^−1^ was due to the hydrogen bond formation between CBZ molecule and material. Another shift was observed from 1209 to 1221 cm^−1^ owing to interactions between sulfur and amino groups or oxygen atom of CBZ molecule. Similar findings were observed for kCAR-TDI (Figure 2d). 

For DCF adsorption, in the case of iCAR-TDI (Figure 2e), a shift from 3260 to 3266 cm^−1^ was due to hydrogen bond formation between CBZ molecule and material. Analogous were the observations for kappa-modified carrageenan and recorded shifts are presenting in corresponding figures.

**Figure 3:**
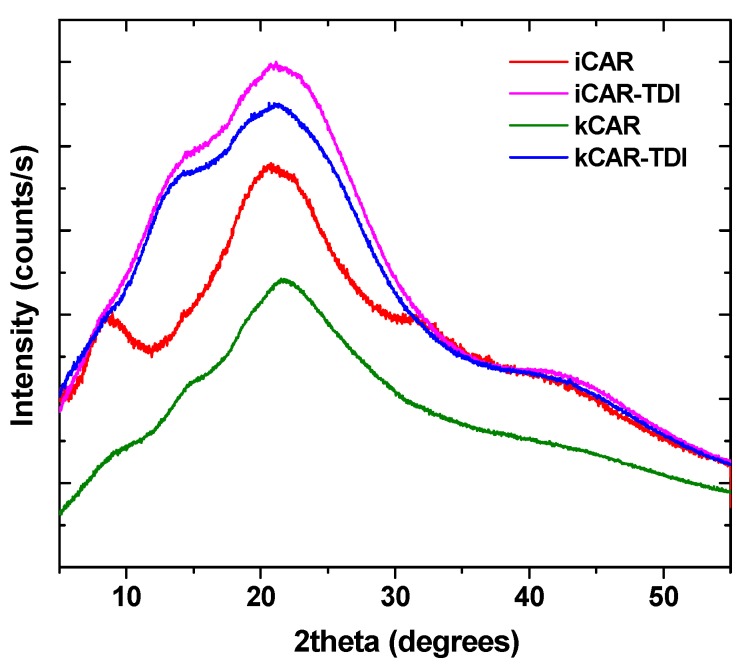
X-ray diffraction (XRD) patterns of iCAR and kCAR before and after TDI-modification.

XRD was used to examine any changes to the physical state of carrageenans (Figure 3). As was foundin a previous study [33], carrageenans show a wide broad peak indicating their amorphous state. The addition of –NCO– groups did not affect the amorphous state and the resulting cross-linked carrageenans also had a wide broad peak.

**Figure 4:**
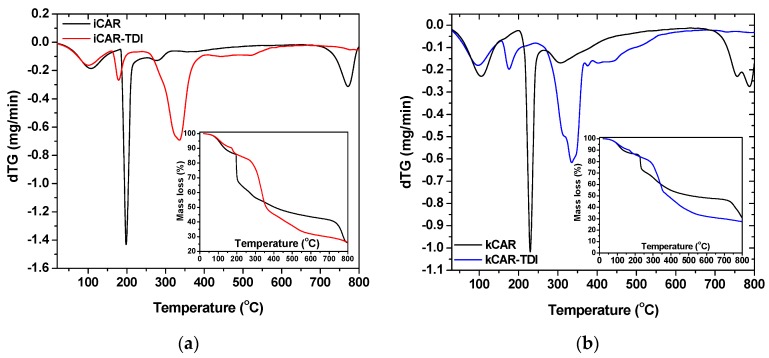
Differential Thermal Analysis (DTG) and Thermal Gravimetric Analysis (TGA) curved for: (**a**) iCAR; and (**b**) kCAR.

To assess the thermal stability of the modified carrageenans, TGA was carried out (Figure 4). As can be seen, thermal degradation profile of –NCO– functionalized (TDI) carrageenan turned out to be quite different compared to that of neat carrageenans. The modified also showed reduced thermal degradation stability due to –NCO– groups. Similar observations were made in previous studies concerning cross-linked chitin and chitosan with HDMI [33,34]. 

Moreover, kCAR showed to be more resistant to thermal stability than iCAR. Both carrageenans showed three decomposition stages (black lines in insets of Figure 4a,b): (i) an initial stage attributed to water evaporation and lasted at 100 °C; (ii) a main decomposition stage due to degradation of the saccharide structure of the molecule, to the dehydration of saccharide rings and decomposition of deacetylated carrageenan units; and (iii) a third stage owing to the acetylated part of the molecule. As was mentioned previously, all samples (modified and non-modified) showed an initial mass loss until 100 °C, which can be reasonably explained by the evaporation of water. DTG curves showed that decomposition for net carrageenans exhibited three stages.

On the other hand, four decomposition stages were observed for the modified carrageenans (red and blue lines in insets of Figure 4a,b): (i) the initial stage of water evaporation; (ii) a second stage owing to –NCO– segments loss; (iii) a third stage of the degradation of the saccharide structure of molecule, the dehydration of saccharide rings and decomposition of deacetylated carrageenan units; and (iv) a fourth stage owed to the acetylated part of the molecule. As was previously reported, modified carrageenans showed reduced thermal stability with iota-modified carrageenan showing the lowest one. A closer observation at the third stage of DTG curves showed that two different decomposition processes might occur during decomposition.

### 3.2. Adsorption Evaluation

#### 3.2.1. pH-Effect

**Figure 5:**
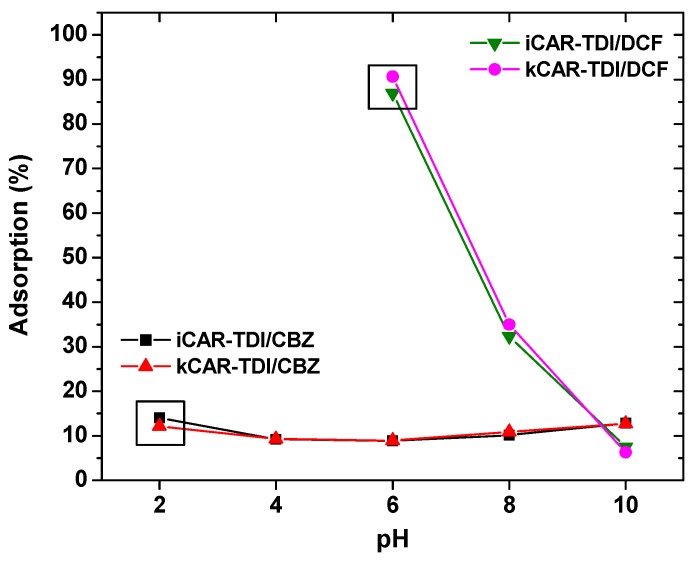
Effect of pH on adsorption of CBZ and DCF onto modified carrageenans (iCAR-TDI and kCAR-TDI).

Solution pH is one of the important parameters affecting the properties of analytes and adsorbents, and therefore the mechanism of aqueous phase adsorption. In other words, the state (cationic/neutral/anionic) of the analyte and the functional groups present/created on the adsorbent are directly related to the corresponding p*K*a values and solution pH. To study the effect of solution pH on the CBZ and DCF adsorption, the initial pH values of drug solutions were varied in the range of 2–10 and 6–10, respectively. In the case of CBZ, the adsorption efficiency does not change in a marked way in the tested solutions (Figure 5). 

This tendency of adsorption may be explained by the fact that, in this pH range, the dominant species of CBZ was neutral molecules (p*K*a 2.3 and 13.9) without charges and thus electrostatic interaction could be ignored in this study. The binding of CBZ onto modified carrageenans is probably controlled by hydrogen bond interactions between hydrogen bonding donor groups (i.e., –NH_2_) and O-donor groups (i.e., –OH, –OSO_3_^−^). At acidic conditions (pH = 2) hydrogen bonding between phenolic OH in carrageenans and carbonyl group (–C=O) in CBZ may be likely dominated. At alkaline conditions, the NH_2_ functional group in CBZ can interact with oxygen-containing functional groups of carrageenans, such as OH and –OSO_3_^−^ functional groups, through hydrogen bonding. H-bond binding mechanisms have been proposed for adsorption of CBZ by other authors using different polymer and porous materials [35,36].

**Figure 6:**
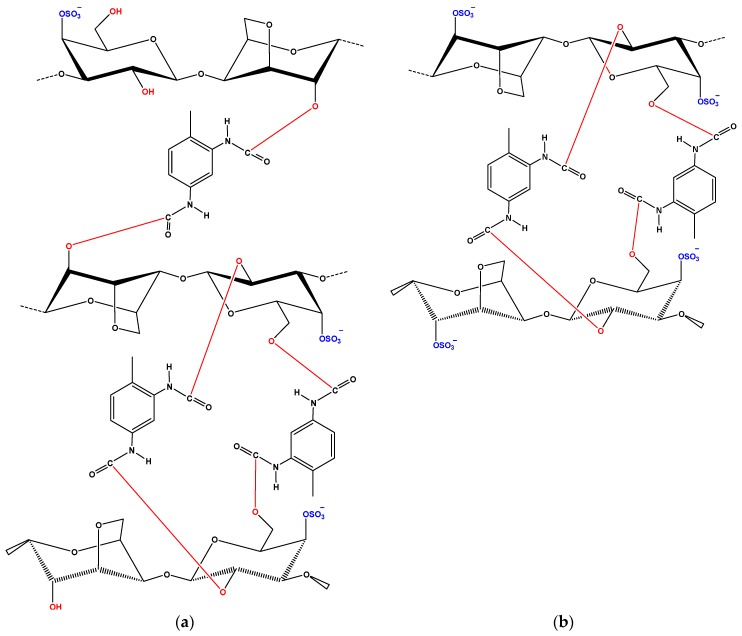
Structures of: (**a**) kCAR-TDI; and (**b**) iCAR-TDI.

In the case of DCF adsorption, the pH range 6–10 was evaluated in order to avoid its precipitation at acidic conditions. DCF is very water soluble in neutral-alkaline medium (50 g/L), but has low solubility (23.7 mg/L) at pH below p*K*a value [37]. The results demonstrated that the adsorption decreased with increasing pH values, due to the possible electrostatic repulsion between the negative surface of modified carrageenans and the anionic DCF at pH > 6 (p*K*a of DCF is 4.15). Similarly to CBZ, hydrogen bonding was likely the predominant adsorption mechanisms of DCF on the surface of modified carrageenans. The DCF molecule has one H-bond donor and one acceptor sites (originating from the –COOH group). On the other hand, modified carrageenans have several acidic (O-/H-containing) functional groups. Therefore, DCF may interact with the carrageenan surface through H-bond formation. The carrageenans can be considered as a H-donor due to the phenol on its surface. It can be suggested that the most probable ways of H-bond formation between the phenolic OH of carrageenans and DCF are those shown in Figure 6. The effects of sulfonate groups on the adsorption of DCF have not debated and were considered steady in the studied pH range, because of the lower p*K*a of –OSO_3_^−^ functional groups and their anionic forms in the studied pH range (pH = 6–10) [38]. This statement was further supported by the FTIR spectra (Figure 2). Furthermore, the H-bond interaction between the phenolic H-atom of carrageenans and the O-atoms of DCF can overcome the possible electrostatic repulsion between the negative surface charges (–COO^−^ of DCF and negative surface of carrageenans) at pH > 6.0 [39].

Based on the aforementioned comments, the pH = 2 and pH = 10 for CBZ and pH = 6 for DCF, were selected as the desirable values for further examination in kinetic and isothermal studies.

#### 3.2.2. Isotherms

One of the most important things regarding adsorption evaluation is the determination of the maximum theoretical adsorption capacity (*Q_max_*). For this reason, Langmuir–Freundlich (L–F) (Equation (2)) isotherm equation [40] was applied to the experimental equilibrium points to fit them.

(2) $$Q_{e}=\frac{Q_{max}K_{LF}{\left(C_{e}\right)}^{1/b}}{1+K_{LF}{\left(C_{e}\right)}^{1/b}}$$

where *Q_e_* (mg/g) is the equilibrium drug concentration in the solid phase; *Q_max_* (mg/g) is the maximum amount of adsorption; *K_LF_* (L/mg)^1/b^ is the L–F constant; and b (dimensionless) is the L–F heterogeneity constant.

The selection of L–F equation and not Langmuir [41] or Freundlich [42] models was based on the fact that, recently, L–F is considered to be the most widely most used isotherm model presenting the most successful/accurate fitting.

The equilibrium amount in the solid phase (*Q_e_*, mg/g) was calculated according to the following equation (where *C*_0_ and *C_e_* (mg/L) are the initial and equilibrium concentrations of drugs, respectively; *V* (L) is the volume of aqueous solution; and *m* (g) is the mass of carrageenans used):

(3) $$Q_{e}=\frac{\left(C_{0}-C_{e}\right)V}{m}$$

**Figure 7:**
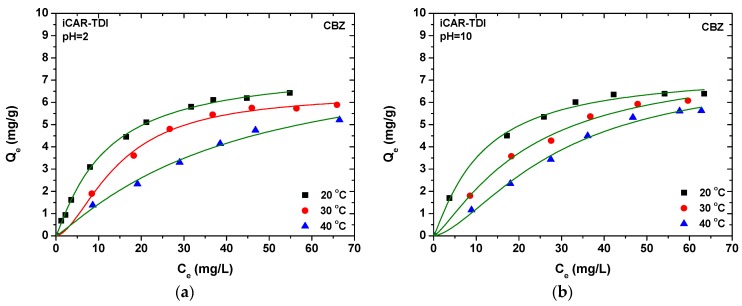

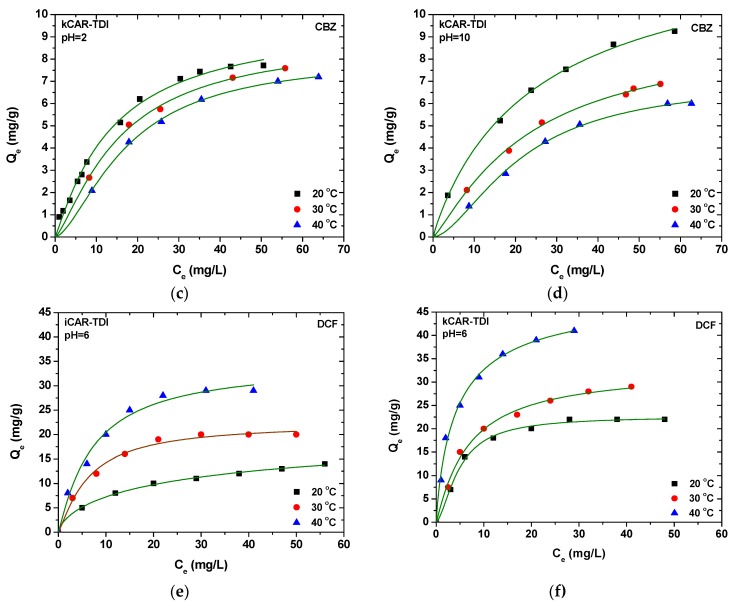
Isotherms for the adsorption of CBZ and DCF onto modified carrageenans. (**a**) Removal of CBZ at pH = 2 by iCAR-TDI; (**b**) Removal of CBZ at pH = 10 by iCAR-TDI; (**c**) Removal of CBZ at pH = 2 by kCAR-TDI; (**d**) Removal of CBZ at pH = 10 by kCAR-TDI; (**e**) Removal of DCF at pH = 6 by iCAR-TDI; (**f**) Removal of DCF at pH = 6 by kCAR-TDI.

In addition, the equilibrium and temperature effect was also studied, as presented in Table 2 and Figure 7. All adsorbents indicate the same behavior; increasing the temperature from 20 to 30 °C, an increase of the adsorption capacity is observed, but a more drastic for the increase from 30 to 40 °C. The same behavior is observed for both CBZ and DCF. At first glance, the increase of temperature influenced more the adsorption of DCF than CBZ. In particular, iCAR-TDI enhances its *Q_max_* for CBZ removal (at pH = 2) from 7.59 mg/g at 20 °C to 7.72 mg/g at 30 °C, and finally 8.39 mg/g at 40 °C. Similar adsorption behavior is revealed for the same combination of carrageenan and CBZ at pH = 10 (from 7.44 mg/g at 20 °C to 7.89 mg/g at 30 °C, and finally 8.51 mg/g at 40 °C). In the case of kCAR-TDI, the temperature effect was also slight (pH = 2: from 9.87 mg/g at 20 °C to 11.12 mg/g at 40 °C; pH = 10: from 7.07 mg/g at 20 °C to 13.78 mg/g at 40 °C). The latter confirms that these adsorbents cannot be substantially influenced by low temperatures (20 and 30 °C), but, at higher temperature (40 °C), the phenomenon becomes more intense. All of the above are listed in Table 1.

### 3.3. Optimization of D-SPE Methodology by Central Composite Design

In extraction procedures, the optimization step is very important to increase the extraction efficiency. In the present study, the pH and the temperature of the solution have been well studied for the adsorption experiments. Desorption solvents were selected primarily using one variable at a time, while, for the other variables, a response surface methodology (RSM) using a central composite design (CCD) was applied for searching the optimal experimental conditions for both analytes. RSM is a multivariate optimization procedure which helps us to find out the optimized condition with the least number of experiments. Based on the higher adsorption capacity for both compounds, kCAR-TDI material was used for the development and optimization of the D-SPE analytical methodology.

#### 3.3.1. Desorption Solvents

To obtain reliable and reproducible analytical results and a high enrichment factor, the eluent for the D-SPE procedure must have high affinity towards the target analytes than the sorbent. In this study, three eluents including methanol, acetonitrile and acetone were tested for elution of DCF and CBZ from the sorbents, according to the principles of green chemistry, to avoid halogenated solvents. When 1 mL of these desorption solvent was evaluated, it was observed that methanol and acetonitrile resulted in higher recoveries than acetone. Ultimately, methanol was chosen as the eluting solvents due to its better extraction efficiency (increase of 5–10% compared to ACN), lower toxicity and cost.

#### 3.3.2. Central Composite Design

The approach of studying one factor at a time cannot describe adequately the importance of certain factors on the extraction process, because interactions between factors are not considered. In this light, to evaluate the combined effects and interactions of extraction time (A), sorbent mass (B) and eluent volume (C), the D-SPE process was further assessed by experimental design and RSM. The second order response surface, which is modeled on the resultsobtained from CCD experiments,can be expressed as:

$$\begin{array}{ll} R_{1} & =+99.97+\left(16.10\times A\right)+\left(10.07\times B\right)+\left(6.84\times C\right)+\left(6.00\times A\times B\right)+\left(2.00\times A\times C\right)+\left(0.00\times B\times C\right)\\ & -13.84A^{2}-11.72B^{2}-11.01C^{2} \end{array}$$

$$\begin{array}{ll} R_{2} & =+92.94+\left(14.54\times A\right)+\left(8.93\times B\right)+\left(8.09\times C\right)+\left(10.37\times A\times B\right)+\left(5.13\times A\times C\right)+\left(1.62\times B\times C\right)\\ & -14.95A^{2}-14.06B^{2}-12.29C^{2} \end{array}$$

**Figure 8:**
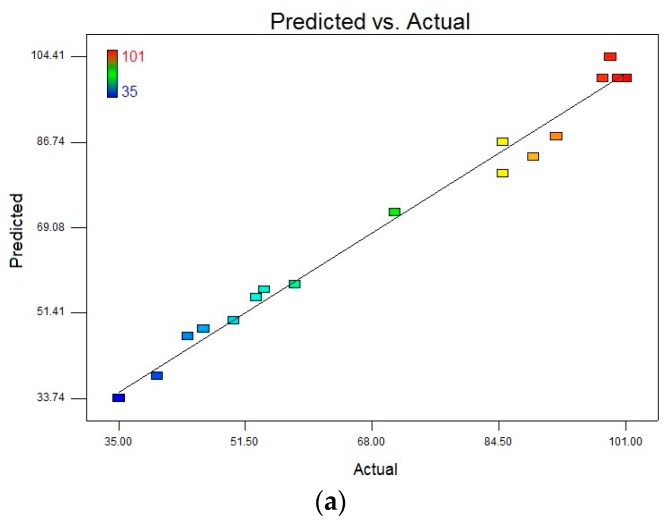

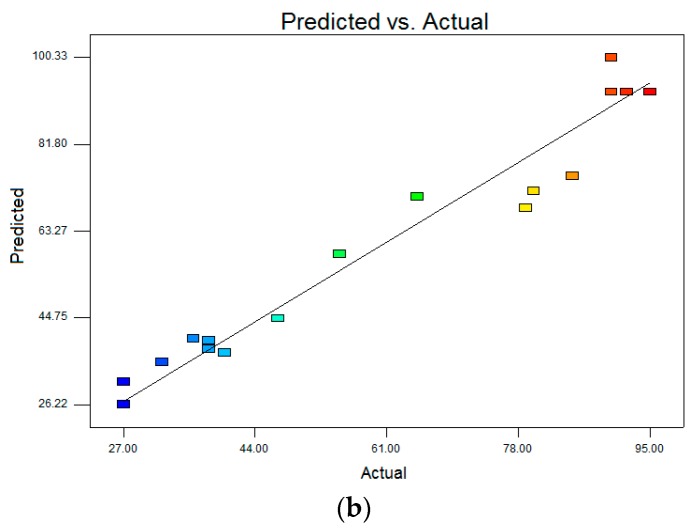
(**a**) The observed responses vs. the predicted responses for DCF; and (**b**) the observed responses vs. the predicted responses for CBZ.

Following to fitting the second order polynomial equation with the actual data of both responses of interest (i.e., DCF (*R*_1_) and CBZ (*R*_2_)), two multiple regression analyses were separately proposed in terms of all the 17 possible combinations of three independent criteria. To test the significance and adequacy of the model, the analysis of variance (ANOVA) was performed for each response and the obtained results are given in Table 4. The statistical significance of the model equations was evaluated by the F-test and *p*-value. According to the results, the high F-values (55.55 for DCF and 15.34 for CBZ) and small *p*-values (<0.0001 for DCF and 0.0008 for CBZ value, both *p*-values < 0.01) suggested that the regression models obtained are highly significant. The goodness of fit of regression model was carried out by determination coefficient (*R*^2^) and adjusted determination coefficient (*R*^2^_adj_). *R*^2^ values computed as 0.9862 for DCF and 0.9517 for CBZ, indicating the goodness-of-fit of the proposed models. Similarly, the values of Adj *R*^2^ for both *R*_1_ and *R*_2_ were reasonably close to 1 (0.9684 for DCF and 0.8897 for CBZ), corroborating a high degree of correlation between the experimental and predicted values. Moreover, the high values of Adequate precision (DCF 21.578 and CBZ 11.323), which measures the signal to noise of the model, indicated a very high degree of precision and a good deal of reliability of the experimental values. This means that the model can be used to navigate the design space. The correlation between observed and predicted values is given in Figure 8. The points are placed very closely to the diagonal line, indicating low discrepancies between them.

Results from the CCD experiments, as shown in Table 4 and Table 5, indicate that the extraction of both analytes depends significantly on all the studied variables, having either negative or positive effects. In particular, the linear terms of extraction time (A), sorbent mass (B) and eluent volume (C), and their quadratic terms A^2^, B^2^ and C^2^, have significant effects on the extraction efficiency of both analytes at 5% significance level (95% confidence interval) as *p*-value for all these terms is less than 0.05. On the other hand, only one considerable interaction effect was established between the extraction time and the sorbent mass for both analytes. The remaining two interaction terms are non-significant as *p*-values for them are more than 0.05%. However, the non-significant terms were not eliminated from the model, since we are interested for the overall effect of the coefficients on the response surface.

**Figure 9:**
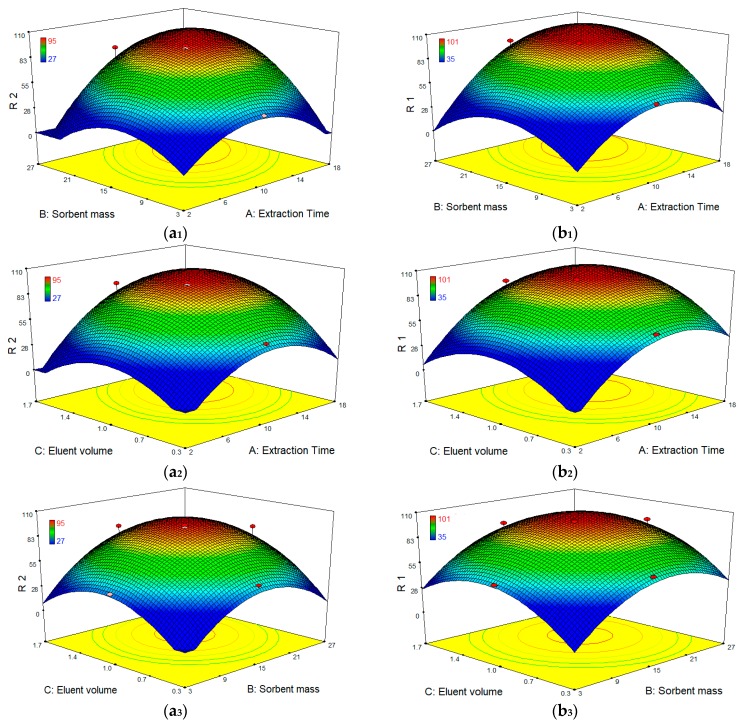
(**a_1_**–**a_3_**) Response surface plots (3D) for the effect of independent variables of D-SPE on to CBZ recovery; and (**b_1_**–**b_3_**) response surface plots (3D) for the effect of independent variables of D-SPE on to DCF recovery.

After identifying the most significant parameters, RSM was used to find optimum condition for the best extraction efficiency. Three-dimensional surface plots (Figure 9) show the interactive effects of extraction time, sorbent mass and eluent volume on the extraction efficiency of both compounds. These plots depict the influence of any two independent variables on the response and the maximum value for each variable can be obtained. According to the results, when eluent volume is fixed at level 0, the extraction efficiency of both compounds is increased by increasing the sorbent amount, reaching a peak of ~100% for DCF and 95% for CBZ at the point of ~15 mg of the sorbent. Such increment in extraction efficiency appears to be because of the accessibility of higher surface areas together with abundant sorption sites. Meanwhile, sorbent addition beyond an adequate quantity to entirely sorb the accessible molecules of the analytes had no significant impact on further improvement in extraction efficiency, demonstrating unsaturated surface active sites of the polymer sorbent. In addition, an increase of extraction efficiency was observed as the extraction time increased up to 10–12 min and then reached a plateau. On the other hand, the extraction efficiency was nearly associated with an enhancement of elution volume from 0.3 to 1 mL and then remained almost constant.

Hence, the optimum working conditions to obtain the best response were as: 1 mL for volume of eluting solvent; 15 mg for sorbent amount; and 15 min extraction time. To confirm the model adequacy for predicting maximum extraction efficiency, three replicate experiments were performed at optimal conditions. A mean value of 97 ± 5.0% for DCF and 92 ± 8.0% for CBZ, obtained from real experiments, demonstrate the suitability of the fitted response surface model.

#### 3.3.3. Performance Characteristics Measured in D-SPE Method Validation

The analytical performance of the developed D-SPE procedure using the novel isocyanate modified carrageenan polymer sorbent was evaluated for the pre-concentration of DCF and CBZ. Analytical figures of merit of the method are summarized in Table 6 and Table 7. 

The calculated calibration curves gave a high level of linearity, yielding coefficients (*r*^2^) > 0.991 for both compounds. LOD and LOQ data of analytes for all water samples are in the ranges of 0.042–0.090 μg/L and 0.137–0.298 μg/L, respectively. The precision of the method was accessed by determining relative standard deviations (RSDs) of intra-day and inter-day at three different spiked levels. The results show that the RSDs of intra-day precision are 2.8–13.5%, while that of inter-day precision are 8.5–17.5%.

Uncertainty of the analytical method was also estimated based on in-house validation data according to EURACHEM/CITAC and GUM guide for both compounds at two spiking levels, as was explained in previous works. The relative expanded uncertainty was lower than 40% for both compounds in all matrices.

**Figure 10:**
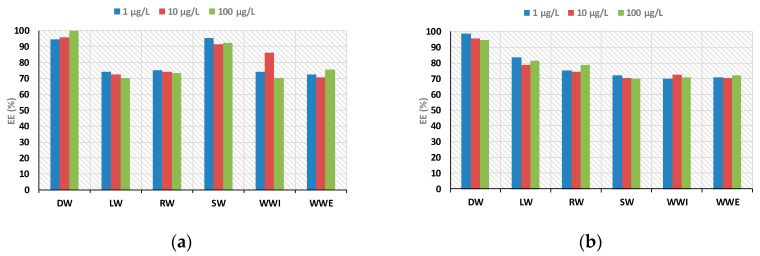
Extraction efficiency (EE, %) of: CBZ (**a**); and DCF (**b**) by D-SPE method in different matrices.

To demonstrate the reliability and versatility of the proposed methodology coupled to LC-MS system for the analysis of real samples, five categories of aqueous samples, including distilled water (DW), river water (RW), sea water (SW), lake water (LW), and influent and effluent wastewaters (WWI and WWE) samples, were analyzed. Recoveries ranged between 70% and 108% for all the matrices, demonstrating the suitability of the proposed method (Figure 10).

## 4. Conclusions

In the present, the application of D-SPE methodology for the pre-concentration of DCF and CBZ has been demonstrated using novel synthesized isocyanate modified carrageenan polymer materials. A central composite design was applied to obtain optimal conditions. Satisfactory precision and accuracy were obtained with the proposed analytical methodology using a small amount of sample. Based on the obtained results, it is anticipated that the proposed method has a great analytical potential for accurate determination of both pharmaceutical compounds in environmental water samples.

## Figures and Tables

**Table 1:** General physicochemical properties of the analytes.

	Carbamazepine	Diclofenac
Molecular Formula	C_15_H_12_N_2_O	C_14_H_11_C_l2_NO_2_
Structure	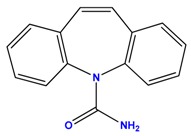	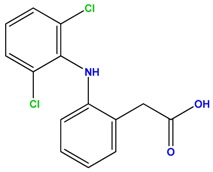
MW (g/mol)	236.27	296.15
Log(K_ow_)	2.45	4.51
p*K*a	2.3; 13.92	4.15
Aqueous solubility (mg/L)	112	2.37

**Table 2:** L–F equilibrium fitting parameters for the adsorption of CBZ and DCF onto modified carrageenans at 20, 30, and 40 °C.

Adsorbent	Drug	pH	*T*	*Q_max_*	*K*_LF_	*b*	*R*^2^
			(°C)	(mg/g)	((L/mg)^1/b^)		
iCAR-TDI	CBZ	2	20	7.59	0.064	0.888	0.999
			30	7.72	0.010	0.585	0.995
			40	8.39	0.015	0.883	0.991
		10	20	7.44	0.062	0.862	0.995
			30	7.89	0.019	0.775	0.994
			40	8.51	0.005	0.640	0.994
kCAR-TDI	CBZ	2	20	9.87	0.054	0.898	0.995
			30	10.99	0.026	0.755	0.998
			40	11.12	0.011	0.631	0.999
		10	20	7.07	0.045	1.061	0.998
			30	9.27	0.022	0.826	0.998
			40	13.78	0.006	0.593	0.998
iCAR-TDI	DCF	6	20	23.41	0.095	1.485	0.998
			30	27.97	0.111	0.835	0.991
			40	37.58	0.115	0.908	0.986
kCAR-TDI		6	20	22.66	0.083	0.628	0.996
			30	35.81	0.128	0.928	0.994
			40	49.29	0.266	1.153	0.994

**Table 3:** Experimental data for CBZ from CCD.

**Standard Order**	**Run**	**Factor 1**	**Factor 2**	**Factor 3**	**Recovery**	
		**A: Extraction Time**	**B: Sorbent Mass**	**C: Eluent Volume**	***R*_1_ DCF**	***R*_2_ CBZ**
		**(min)**	**(mg)**	**(mL)**	**(%)**	**(%)**
12	1	10	27	1.0	89	79
8	2	15	22	1.4	99	90
1	3	5	8	0.6	40	40
4	4	15	22	0.6	85	65
2	5	15	8	0.6	53	32
11	6	10	3	1.0	50	38
9	7	1.6	15	1.0	35	27
16	8	10	15	1.0	98	92
7	9	5	22	1.4	54	36
15	10	10	15	1.0	101	95
5	11	5	8	1.4	46	38
13	12	10	15	0.3	58	47
14	13	10	15	1.7	85	80
3	14	5	22	0.6	44	27
6	15	15	8	1.4	71	55
10	16	18	15	1.0	92	85
17	17	10	15	1.0	100	90
**Factor**	**Name**	**Units**	**Low Actual (Coded)**	**High Actual (Coded)**	**Mean**	**Standard Deviation**
A	Extraction Time	min	5	15	10	4.481
B	Sorbent mass	mg	8	22	15	6.274
C	Eluent volume	ml	0.6	1.40	1	0.359

**Table 4:** ANOVA test for CBZ by CCD.

Source	Sum of Squares	df ^a^	Mean Square	F	*p*-Value	Significant
				Value ^b^	Prob > F ^c^	
Model	10,049.37	9	1116.60	15.34	0.0008	yes
A-Extraction Time	2886.44	1	2886.44	39.64	0.0004	
B-Sorbent mass	1089.03	1	1089.03	14.96	0.0062	
C-Eluent volume	894.06	1	894.06	12.28	0.0099	
AB	861.13	1	861.13	11.83	0.0109	
AC	210.13	1	210.13	2.89	0.1332	
BC	21.12	1	21.12	0.29	0.6068	
A^2^	2518.01	1	2518.01	34.58	0.0006	
B^2^	2228.98	1	2228.98	30.61	0.0009	
C^2^	1703.76	1	1703.76	23.40	0.0019	
Residual	509.68	7	72.81			
Lack of Fit	497.02	5	99.40	15.70	0.0610	no
Pure Error	12.67	2	6.33			
Cor Total	10,559.06	16				

^a^ Degree of freedom; ^b^ Test for comparing model variance with residual (error) variance; ^c^ Probability of seeing the observed F value if the null hypothesis is true.

**Table 5:** ANOVA test for DCF by CCD.

Source	Sum of Squares	df ^a^	Mean Square	F	*p*-Value	Significant
				Value ^b^	Prob > F ^c^	
Model	9114.50	9	1012.72	55.55	<0.0001	yes
A-Extraction Time	3539.57	1	3539.57	194.15	<0.0001	
B-Sorbent mass	1386.19	1	1386.19	76.03	<0.0001	
C-Eluent volume	638.88	1	638.88	35.04	0.0006	
AB	288.00	1	288.00	15.80	0.0054	
AC	32.00	1	32.00	1.76	0.2268	
BC	0.00	1	0.00	0.00	1.0000	
A^2^	2160.21	1	2160.21	118.49	<0.0001	
B^2^	1548.86	1	1548.86	84.95	<0.0001	
C^2^	1367.62	1	1367.62	75.01	<0.0001	
Residual	127.62	7	18.23			
Lack of Fit	122.95	5	24.59	10.54	0.0889	no
Pure Error	4.67	2	2.33			
Cor Total	9242.12	16				

^a^ Degree of freedom; ^b^ Test for comparing model variance with residual (error) variance; ^c^ Probability of seeing the observed F value if the null hypothesis is true.

**Table 6:** Analytical performance of D-SPE for CBZ in different matrices.

	Spiking Level (μg/L)	DW	LW	SW	RW	WWI	WWE
LOD (μg/L)	-	0.042	0.046	0.043	0.047	0.062	0.047
LOQ (μg/L)	-	0.137	0.151	0.142	0.154	0.205	0.154
RSD_r_ (%)							
(*n* = 5)	1	7.2	11.2	13.1	9.6	12.1	11.2
	10	5.1	9.1	10.9	7.7	11.2	9.8
	100	3.7	8.8	9.2	7.5	10.7	9.0
RSD_R_ (%)							
(*n* = 5)	1	10.2	15.3	16.1	14.1	17.5	17.0
	10	9.8	13.7	14.8	12.8	15.2	14.9
	100	9.1	10.2	14.1	11.4	14.0	12.3
U (%)	1	33.92	33.2	33.41	34.86	39.91	37.19
(*k* = 2, confidence level 95%)	10	15.96	16.68	17.24	17.03	19.88	18.56
	100	9.91	10.84	12.17	11.28	13.62	12.22

**Table 7:** Analytical performance of D-SPE for DCF in different matrices.

	Spiking Level (μg/L)	DW	LW	SW	RW	WWI	WWE
LOD (μg/L)	-	0.060	0.067	0.063	0.068	0.090	0.068
LOQ (μg/L)	-	0.199	0.220	0.207	0.225	0.298	0.225
RSD_r_ (%)							
(*n* = 5)	1	5.1	11.5	12.8	8.0	9.7	10.5
	10	3.9	8.9	10.5	7.1	10.2	9.3
	100	2.8	8.1	10.0	6.8	9.5	8.8
RSD_R_ (%)							
(*n* = 5)	1	9.6	14.6	15.9	13.4	15.6	14.1
	10	8.5	12.4	13.9	11.8	13.6	12.9
	100	9.5	11.4	12.7	10.2	12.9	11.8
U (%)	1	24.51	27.49	23.05	25.28	36.57	28.42
(*k* = 2, confidence level 95%)	10	11.67	14.04	13.08	12.94	18.09	14.68
	100	8.06	10.0	10.25	8.99	12.44	10.51
